# Molecular modeling of CO_2_ affecting competitive adsorption within anthracite coal

**DOI:** 10.1038/s41598-024-58483-z

**Published:** 2024-03-30

**Authors:** Lin Hong, Jiaxing Lin, Dameng Gao, Dan Zheng

**Affiliations:** 1 College of Safety Science & Engineering, Liaoning Technical University, No. 188 Longwan South Street, Huludao, 125105 Liaoning China; 2 Key Laboratory of Mine Thermodynamic Disaster & Control of Ministry of Education, Liaoning Technical University, Huludao, 125105 Liaoning China; 3 Institute of Mine Safety Technology, China Academy of Safety Science and Technology, Beijing, 100012 China

**Keywords:** Coal, Competitive adsorption, Energy distribution, Grand canonical ensemble Monte Carlo, Molecular dynamics, Energy science and technology, Engineering

## Abstract

This study aimed to investigate the adsorption properties of CO_2_, CH_4_, and N_2_ on anthracite. A molecular structural model of anthracite (C_208_H_162_O_12_N_4_) was established. Simulations were performed for the adsorption properties of single-component and multi-component gases at various temperatures, pressures, and gas ratios. The grand canonical ensemble Monte Carlo approach based on molecular mechanics and dynamics theories was used to perform the simulations. The results showed that the isotherms for the adsorption of single-component CO_2_, CH_4_, and N_2_ followed the Langmuir formula, and the CO_2_ adsorption isotherm growth gradient was negatively correlated with pressure but positively correlated with temperature. When the CO_2_ injection in the gas mixture was increased from 1 to 3% for the multi-component gas adsorption, the proportion of CO_2_ adsorption rose from 1/3 to 2/3, indicating that CO_2_ has a competing-adsorption advantage. The CO_2_ adsorption decreased faster with increasing temperature, indicating that the sensitivity of CO_2_ to temperature is stronger than that of CH_4_ and N_2_. The adsorbent potential energies of CO_2_, CH_4_, and N_2_ diminished with rising temperature in the following order: CO_2_ < CH_4_ < N_2_.

## Introduction

Technologies for infusing CO_2_ or CO_2_–N_2_ mixtures into coal seams to replace CH_4_ have gradually matured in recent years, improving CH_4_ extraction rates and enabling the sequestration of large amounts of CO_2_^1–4^. These technologies primarily utilize the different adsorption abilities of CO_2_, CH_4_, and N_2_ on coal to improve the CH_4_ extraction rate.

Gases in coal are mainly adsorbed physically. N_2_ and CO_2_ have a wide range of sources, where N_2_ accounts for approximately 78% of the atmosphere while CO_2_ is mainly generated through coal oxidation and combustion^5^. Scholars around the world have conducted multiple gas adsorption experiments^6–8^ and Monte Carlo simulation studies^9–11^ to investigate the adsorption characteristics of different gases on coal. The impact of temperature on gas adsorption has been investigated using the high-pressure volumetric method, which showed that low temperatures hinder gas adsorption on coal^12,13^. Li et al.^14^ proposed the theory of the competitive adsorption of multiple gases using the low-temperature nitrogen adsorption method. Zhu et al.^15^ experimentally verified that the maximum adsorption capacities of CO_2_, CH_4_, and N_2_ on anthracite exceed those on bituminous coal at different temperatures. Xiao et al.^16,17^ established the relationship between the coal adsorption capacity and temperature for CO_2_, CH_4_, and N_2_. Cheng et al.^18,19^ applied the grand canonical ensemble Monte Carlo method to investigate the adsorption properties of CO_2_/N_2_ on coal. Similarly, Wu et al.^20,21^ investigated the microscopic mechanism of the competitive adsorption of coal-fired flue gas on coal. The research revealed the competitive adsorption behavior during the gas adsorption. Qu et al.^22^ developed a macromolecular model of coal and used the grand canonical Monte Carlo (GCMC) and molecular dynamics (MD) molecular simulations to reveal the microscopic mechanisms of CO_2_, O_2_, and CH_4_ adsorption and diffusion on coal molecules at different temperatures, pressures, and molar fractions. Gao et al.^23^ analyzed the adsorption capacity of CO_2_/CH_4_/N_2_/H_2_O on different molecular models of brown coal using molecular simulation. Wang et al.^24^ used molecular simulation to examine the adsorption behavior of CH_4_/CO_2_ on oxidized coal deposits. Yu et al.^25^ conducted experiments and simulations to examine the competitive adsorption mechanism of CO_2_ and CH_4_ on low-rank coal mirror groups. Bai et al.^26^ performed a molecular simulation to investigate the kinetic mechanism of CO_2_ and N_2_ replacement of CH_4_. The results showed that CO_2_ and N_2_ mainly replaced CH_4_ gas by occupying the adsorption site. Liu et al.^27^ performed CO_2_-ECBM tests on rectangular coal specimens and monitored the change patterns of pore pressure, gas flow, and outlet concentration with the amount of CO_2_ injected; they found that infusing CO_2_ into the coal body can effectively increase the CH_4_ recovery. Li et al.^28^ proposed a dynamic evolution model of coal permeability, which responded to the ECBM mining process and confirmed the technical feasibility of N_2_-BCEM injection.

Other international scholars have also studied the adsorption properties of gases consisting of single components and multiple components. Using computer molecular simulations, Laurent et al.^29^ investigated the absorbent behaviors of CO_2_ and CH_4_ in micropores. Perera et al.^30^ investigated the adsorption capacity of two gases at three temperatures. Abunowara et al.^31^ analyzed the characteristics of four coal sample adsorption gases in Malaysia using BET, XRD, FESEM, and other techniques, and the coal specimens exhibited a greater adsorption affinity for CO_2_ than for CH_4_ and N_2_. Busch et al.^32^ studied the gas adsorption of binary mixtures at a specific pressure using Langmuir’s method, which provides an essential idea for this current study on the adsorption of ternary gas mixtures.

The studies highlighted above mainly focused on the adsorption characteristics of binary gas systems, confirming the advantage of CO_2_ adsorption but ignoring the adsorption characteristics of ternary gas systems. In this article, we quantitatively analyzed the adsorption behavior of CO_2_, CH_4_, and N_2_ on coal and the main influencing factors. The injection of different ratios of CO_2_, CH_4_, and N_2_ into coal seams was simulated for the study.

## Methodology

### Molecular modeling of anthracite

**Figure 1:**
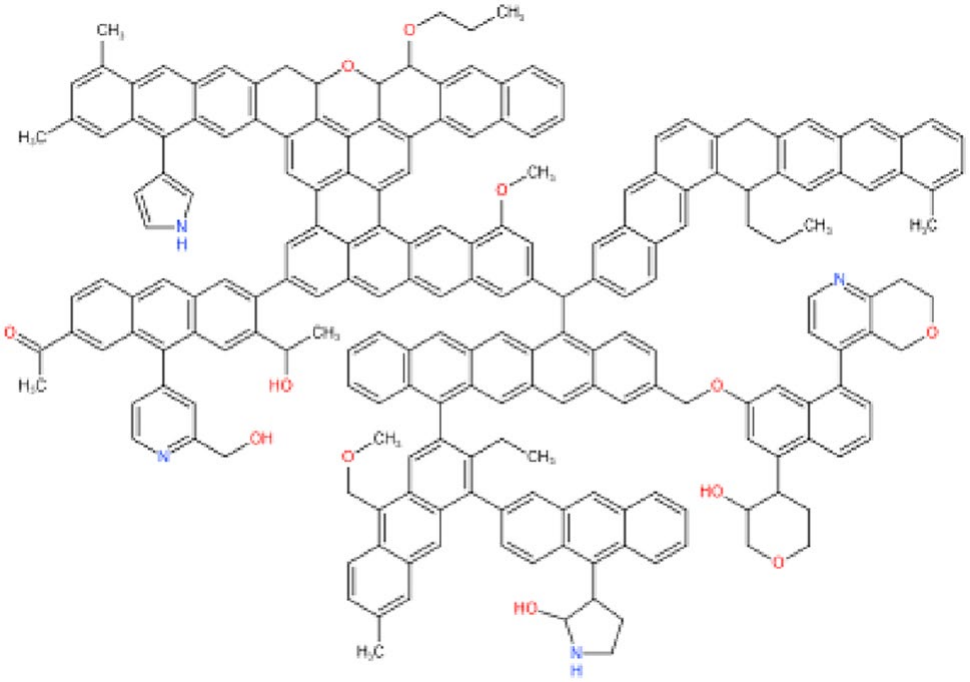
Molecular structure of anthracite.

**Figure 2:**
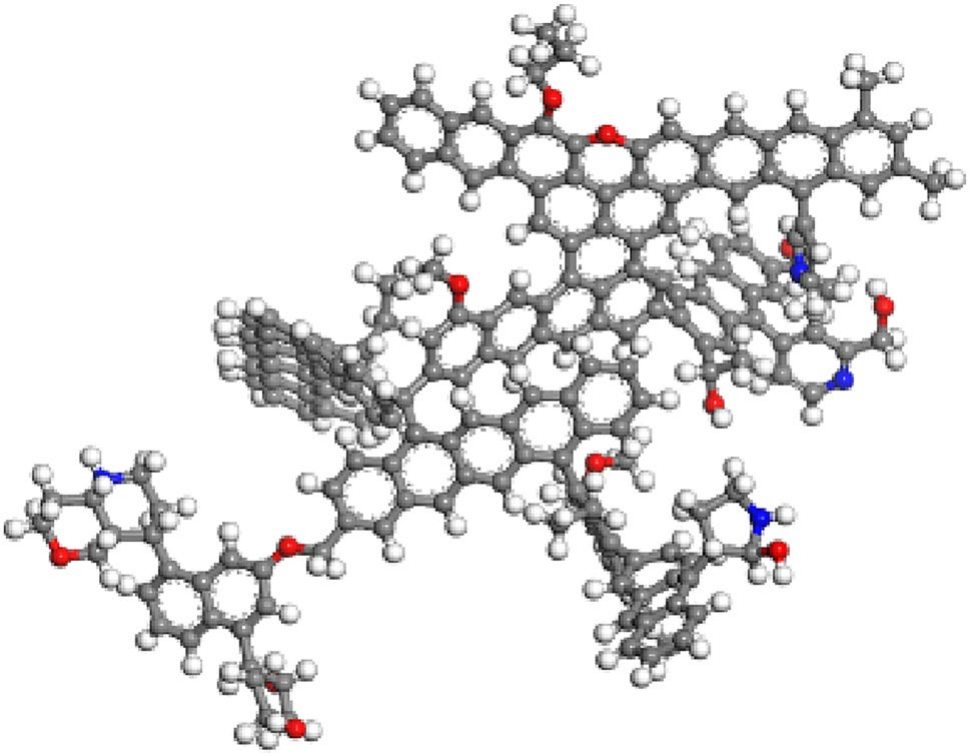
Molecular structural model of anthracite.

**Figure 3:**
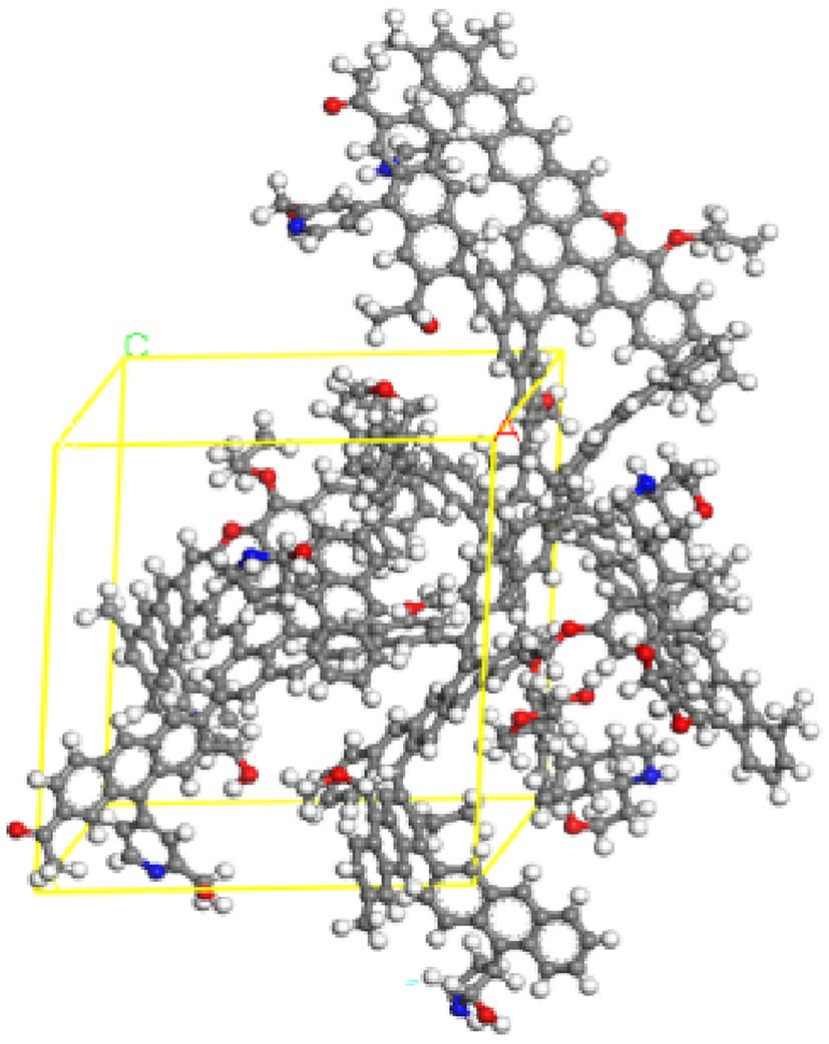
Pore structure model of anthracite.

The anthracite molecule (C_208_H_162_O_12_N_4_)^33^ (illustrated in Fig. 1) was selected to study the adsorption properties of anthracite for CO_2_, CH_4_, and N_2_. Geometry optimization, energy optimization, and simulated annealing were performed on the structure using the Forcite module in Materials Studio, and the optimized energy parameters are shown in Table 1 to obtain the lowest energy conformation, as shown in Fig. 2. The Amorphous cell module was used to put the two optimized anthracite molecular models into the computational cell (a = 18.607 Å, b = 18.607 Å, c = 18.607 Å), as shown in Fig. 3.

**Table 1:** Energy parameters of optimized coal molecular structure.

E _total_ (kcal/mol)	E_V_ (kcal/mol)				E_N_ (kcal/mol)		
	E_B_	E_A_	E_T_	E_I_	E_VAN_	E_E_	E_H_
343.389	107.235	86.263	126.985	3.024	525.086	− 5.337	− 0.0001

*E*_V_ valence energy, *E*_B_ bond energy, *E*_A_ angle energy, *E*_T_ torsion energy, *E*_I_ inversion energy, *E*_N_ nonbond energy, *E*_VAN_ van der Waals energy, *E*_E_ electrostatic energy; *E*_H_ hydrogen bond energy.

### Simulation parameter setting

To simulate the adsorption of CO_2_, CH_4_, and N_2_ on anthracite coal, we used a giant regular ensemble Monte Carlo simulation, selecting the Dreiding force field customized for computational accuracy, Charges selects QEq, the Ewald summation method for electrostatic interactions, and the atom-based method for van der Waals interactions. The temperatures used for the Fixed Pressure Task were 263.15 K, 273.15 K, 283.15 K, 293.15 K, 303.15 K, and 313.15 K, and the pressure ranged from 0.01 to 2 MPa.

## Results and discussion

### Single-component gas adsorption system

**Figure 4:**
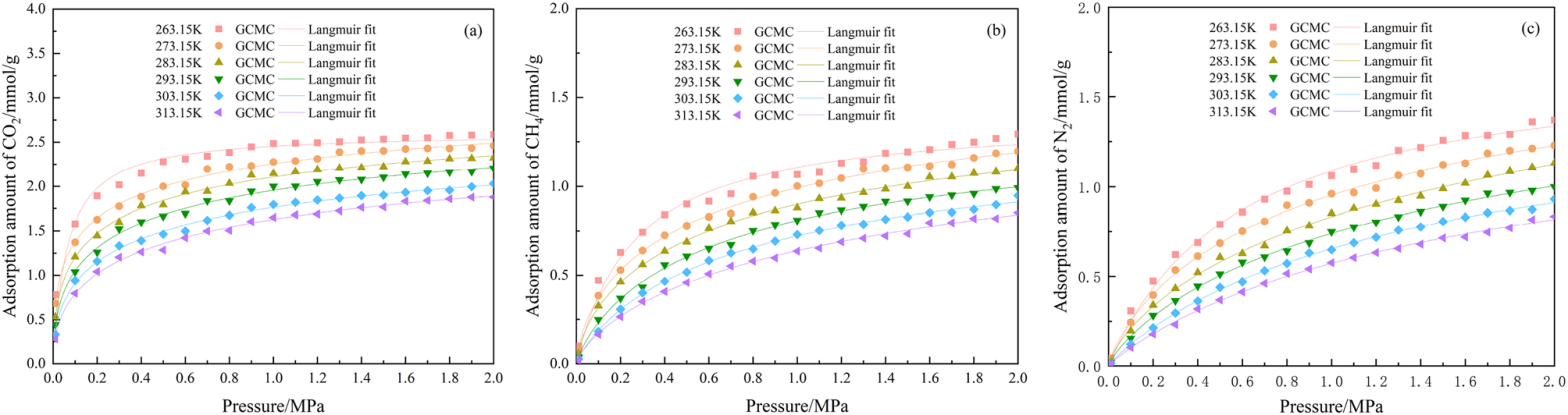
Isothermal adsorption curves for single-component: (**a**) CO_2_, (**b**) CH_4_ and (**c**) N_2_.

**Figure 5:**
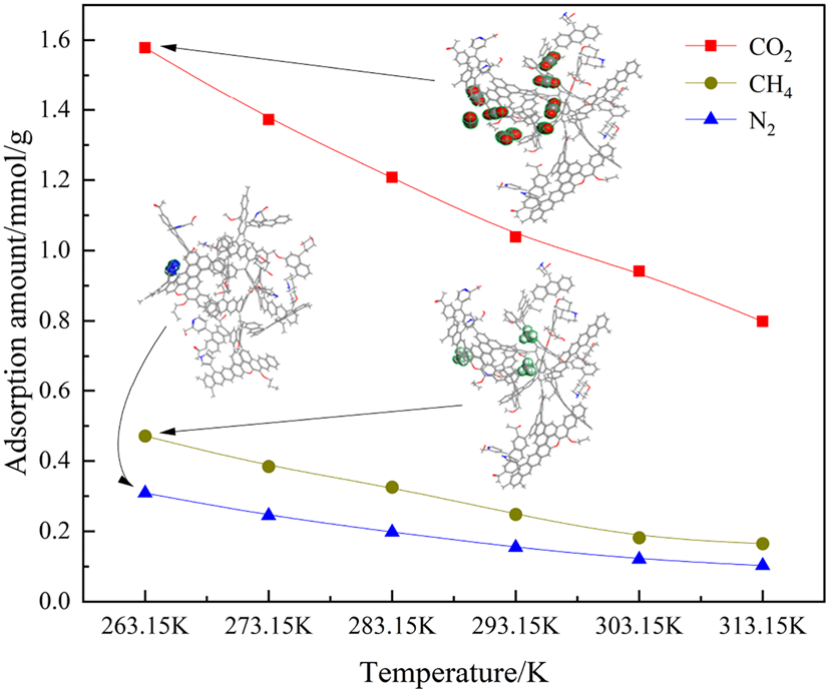
Variation of single-component gas adsorption with temperature at 0.1 MPa.

Figure 4 illustrates the adsorption isotherms of N_2_, CO_2_, and CH_4_, as single-component gases under varying pressures and temperatures. As observed from the figure, at temperatures of 263.15 K, 273.15 K, 283.15 K, 293.15 K, 303.15 K, and 313.15 K and at pressures ranging from 0.01 to 2 MPa, the adsorption sites on the surface of the anthracite coal become more active with increasing temperature. Consequently, CO_2_, CH_4_, and N_2_ are more readily detached from the coal surface, reducing the adsorption capacity. Similarly, Fig. 5 illustrates the variation of the CO_2_, CH_4_, and N_2_ adsorption with temperature at a pressure of 0.1 MPa. At 263.15 K, a considerably larger number of adsorbed CO_2_ molecules are in the anthracite molecular model than the number of CH_4_ and N_2_ molecules. With the increase of pressure from 0.01 MPa to 2 MPa in the system, the adsorption amount of CO_2_, CH_4_ and N_2_ showed an upward trend, indicating that the elevated pressure can promote the adsorption of CO_2_, CH_4_ and N_2_ by anthracite. The magnitude of adsorption at the same pressure is in the following order: CO_2_ > CH_4_ > N_2_. This result can be explained as follows: first, the molecules of the three gases have different equivalent diameters, where the molecular diameter of CO_2_ is 0.33 nm, that of N_2_ is 0.368 nm, and that of CH_4_ is 0.382 nm. Because the diameter of CO_2_ molecule is small, the critical temperature and critical pressure of CO_2_ are larger, which makes the competitive adsorption advantage of CO_2_ stronger than that of CH_4_ and N_2_ in the ternary gas system of CO_2_, CH_4_, and N_2_, and therefore the anthracite molecules preferentially adsorb CO_2_, so it leads to the adsorption amount of CO_2_ is larger than that of CH_4_ and N_2_. Owing to the small difference between the molecular diameters of CH_4_ and N_2_, both have different polarization volume, where that of CH_4_ is 4.48 × 10^–30^ m^3^ and that of N_2_ is 1.53 × 10^–30^ m^3^. The molecules with a higher polarization volume are adsorbed more easily; thus, the amount of CH_4_ adsorbed is larger than that of N_2_. Secondly, the critical temperatures are different, the critical temperatures of CO_2_, CH_4_ and N_2_ are 304 K, 191 K and 126 K. The size of the critical temperatures is CO_2_ > CH_4_ > N_2_. As the critical temperature increases, the gas adsorption on the surface of the coal becomes faster and adsorbs more easily. Third, van der Waals forces also play a role: an increase in the pressure of the system is accompanied by an increase in the van der Waals energy, and the stronger the effect of the van der Waals force, the faster the adsorption. Take a temperature of 263.15 K as an example, the van der Waals energy data released by anthracite adsorption of CO_2_, CH_4_, and N_2_ are shown in Table 2.

**Table 2:** Van der Waals energy released by adsorption of CO_2_, CH_4_ and N_2_ from anthracite coal at pressures of 0.01 MPa and 2 MPa.

Gas	P/(MPa)	E_VAN_/(kcal/mol)
CO_2_	0.01	− 49.447
	2	− 87.397
CH_4_	0.01	− 6.198
	2	− 37.306
N_2_	0.01	0
	2	− 28.859

The isothermal adsorption curves of CO_2_, CH_4_, and N_2_ at different pressure ranges have the form of Langmuir curves. Hence, the Langmuir formula was used to fit the isothermal adsorption curves of CO_2_, CH_4_, and N_2_ for anthracite. The fitting results are presented in Table 3.

**Table 3:** Fitting parameters of the Langmuir model.

Gas	T/K	Anthracite/(0.1–2 MPa)			Anthracite/(0.01–0.1 MPa)		
		k_1_/(mmol/g)	k_2_/(MPa^–1^)	R^2^	k_1_/(mmol/g)	k_2_/(MPa^–1^)	R^2^
CO_2_	263.15 K	15.45930	12.41058	0.97808	10.22002	66.22327	0.94513
	273.15 K	14.83972	8.65084	0.95236	8.68364	60.82048	0.96314
	283.15 K	14.14749	7.24969	0.96037	8.12678	49.34100	0.95666
	293.15 K	13.49824	6.00503	0.96425	7.01259	47.49367	0.97249
	303.15 K	12.34502	5.50192	0.94853	6.84291	32.66120	0.98443
	313.15 K	11.75505	4.62049	0.95514	5.98387	29.65065	0.98381
CH_4_	263.15 K	8.13773	3.74307	0.97177	5.32766	13.39820	0.98118
	273.15 K	7.92619	2.85655	0.98222	3.73444	10.85104	0.98689
	283.15 K	7.44071	2.51863	0.98498	4.35184	7.07093	0.98279
	293.15 K	7.19663	1.94083	0.99350	4.06383	5.81027	0.98742
	303.15 K	6.84858	1.62792	0.99534	2.98200	5.26464	0.98378
	313.15 K	6.49959	1.39296	0.99171	4.67810	2.40749	0.99387
N_2_	263.15 K	10.05641	1.69437	0.99118	4.58182	6.39676	0.99840
	273.15 K	9.33844	1.51337	0.99350	5.15792	3.97161	0.99735
	283.15 K	9.09189	1.18631	0.99201	4.62266	3.30171	0.99848
	293.15 K	8.46873	1.06177	0.99591	5.24324	2.09114	0.99755
	303.15 K	8.43825	0.83123	0.99830	4.58101	1.84693	0.99772
	313.15 K	8.01405	0.71803	0.99745	18.97877	0.32407	0.99886

The fitting results show that the linear correlation coefficient R^2^ was greater than 0.94 in all cases, indicating good fitting and confirming the reliability of the simulated data. The adsorption constants k_1_, k_2_ decrease with increasing temperature; the adsorption amount also decreases, indicating that low temperatures are favorable for CH_4_ adsorption.

Figure 4 shows that the adsorption isotherm growth gradient of CO_2_, CH_4_, and N_2_ within the pressure range of 0.01–0.1 MPa is considerably larger than that within the pressure ranges of 0.1–1 MPa and 1–2 MPa. Therefore, CO_2_ was used as an example to calculate the adsorption isotherm growth gradients of the gases at different temperatures, as shown in Table 4.

**Table 4:** Growth gradient of CO_2_ adsorption isotherms at different temperatures.

P/MPa	T/K					
	263.15	273.15	283.15	293.15	303.15	313.15
0.01–0.1	1.985	2.017	2.262	2.307	2.768	2.809
0.1–1.0	1.468	1.553	1.644	1.757	1.790	1.892
1.1–2.0	0.975	1.000	1.009	1.027	1.029	1.035

At different temperatures, the growth gradients of CO_2_ adsorption isotherms were 1.985–2.809 for pressures of 0.01–0.1 MPa, 1.468–1.892 for pressures of 0.1–1.0 MPa, and 0.975–1.035 for pressures of 1.1–2.0 MPa. These results show that the CO_2_ adsorption isotherm growth gradient is the largest and the adsorption rate is the fastest in the pressure range of 0.01–0.1 MPa. This phenomenon occurs because of the large number of adsorption sites on the anthracite surface. Initially, CO_2_, CH_4_, and N_2_ are easily adsorbed on these sites, resulting in a faster adsorption rate, but the gas concentration increases while the adsorption sites are gradually saturated, causing the adsorption rate to decelerate until it reaches equilibrium. Therefore, the larger the growth gradient of the adsorption isotherm, the faster the adsorption rate. This behavior occurs because the growth gradient represents the rate of gas adsorption; the faster the adsorption rate, the greater the number of adsorption sites on the anthracite surface.

### Multi-component gas adsorption system

#### Adsorption amount

**Figure 6:**
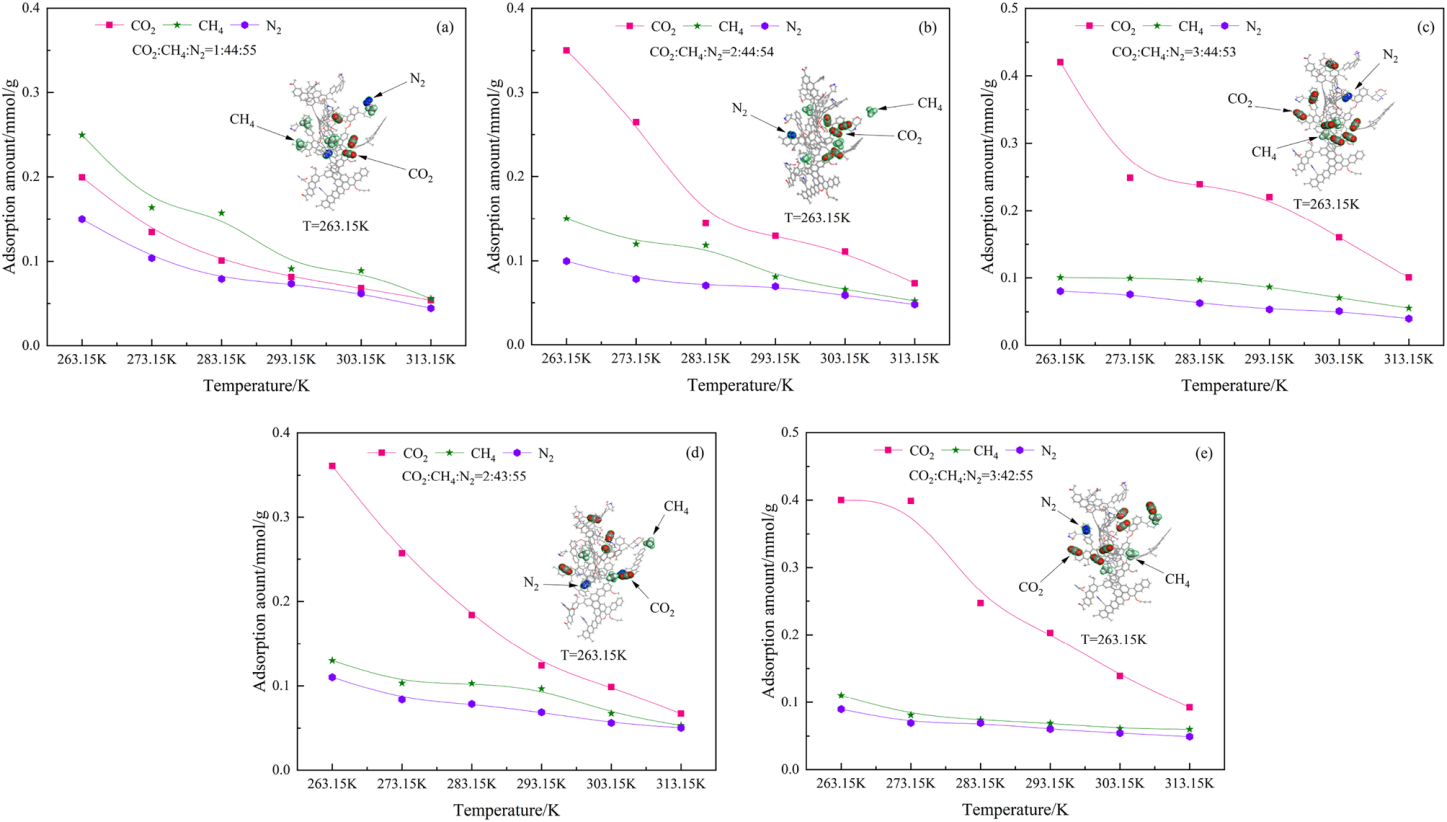
Variation of multi-component gas adsorption amount with temperature at 0.1 MPa.

The single-component gas adsorption shows that CO_2_ has certain adsorption advantages over CH_4_ and N_2_. Different proportions of CO_2_, CH_4_, and N_2_ were added to the anthracite pore model. As the adsorption rate of the gases with pressure ranging from 0.01 to 0.1 MPa is the fastest in the single-component gas adsorption system, the adsorption characteristics of the three gases are discussed for a pressure of 0.1 MPa. The simulation results are shown in Fig. 6.

A comparison of Fig. 6a–c shows that adding the same ratio of CH_4_ and different ratios of CO_2_ and N_2_ to the anthracite molecule with the temperature of 263.15 K, the relative CO_2_ adsorption increases from 0.20 to 0.42 mmol/g as the CO_2_ injection increases from 1 to 3%; The relative adsorption of the same proportion of CH_4_ decreases with the gradual increase in the CO_2_ injection. The relative adsorption of CH_4_ decreases from 0.25 to 0.10 mmol/g while that of N_2_ in different proportions decreases from 0.15 to 0.08 mmol/g. A comparison of the plots in Fig. 6a,d and e shows that at the temperature of 263.15 K, injecting the same proportion of N_2_ and different proportions of CO_2_ and CH_4_ increases the relative adsorption of CO_2_ with increasing amount of injected gas from 0.20 to 0.40 mmol/g. The relative adsorption of CH_4_ decreases from 0.25 to 0.11 mmol/g with decreasing CH_4_ injection, and the relative adsorption of equal proportions of N_2_ decreases from 0.15 to 0.09 mmol/g. These results show that the adsorption sensitivity of CO_2_ is very strong, and the relative CO_2_ adsorption increases rapidly when the CO_2_ injection increases from 1 to 3%, which far exceeds the relative adsorption of CH_4_ and N_2_. Second, the relative adsorption of CH_4_ decreases slowly as CO_2_ injection increases in the system, which is because CO_2_ has certain adsorption advantages and a stronger adsorption capacity than CH_4_. Hence, CO_2_ is preferentially adsorbed, decreasing the relative adsorption of CH_4_. Third, different proportions of N_2_ and equal proportions of N_2_ have less effect on changes in the adsorption amount of the system, indicating that N_2_ adsorption is more stable. The amount of gas adsorbed is affected not only by temperature and gas injections, but also by the adsorption potential. When a gas is adsorbed on the surface of the coal body, the adsorbent is also attracted to the adsorbate; the closer to the surface, the greater the gravitational force, which is the adsorption potential; thus, the adsorption amount is also related to the adsorption potential.

#### Adsorption potential

Polanyi^34^ and Dubinin^35^ proposed the adsorption potential theory. The theory posits that an adsorption potential field encircles a solid, and gas molecules are adsorbed within this field through the influence of attractive forces. Consequently, the connection between the adsorption potential and adsorption amount was used to analyze the adsorption features of three gases: CO_2_, CH_4_, and N_2_.

According to Polanyi, the relationship between adsorption potential and pressure is as follows:

1 $$\varepsilon =RT{\text{ln}}\frac{{P}_{0}}{P}.$$

In this context, ε represents the adsorption potential [J/mol]; R represents the ideal gas constant, taken as 8.3144 J/(mmol·K); T represents the absolute temperature [K]; P0 represents the vapor pressure at saturation of the gas corresponding to temperature T [MPa]; and P represents the pressure [MPa].

According to Dubinin, the formula for calculating P0 is,

2 $${P}_{0}={P}_{c}{\left(\frac{T}{{T}_{C}}\right)}^{2}.$$

In this context, Pc represents the critical pressure; the critical pressures of CO_2_, CH_4_, and N_2_ are taken as 7.38 MPa, 4.60 MPa, and 3.40 MPa, respectively. Tc represents the critical temperature, and the critical temperatures of CO_2_, CH_4_, and N_2_ are taken as 304.13 K, 190.56 K, and 126.20 K, respectively.

**Figure 7:**
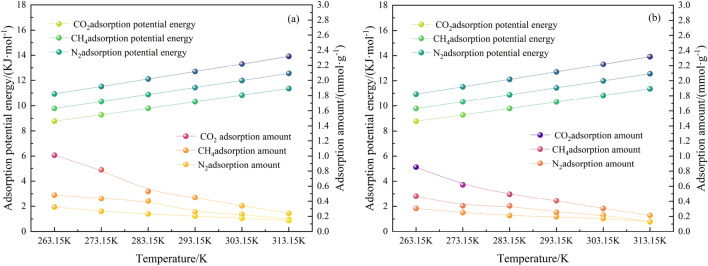
Curves of relationship between adsorption amount and adsorption potential energy of multi-component gases at different temperatures. (**a**) is injected in the ternary gas system with the same proportion of CH_4_, (**b**) is injected in the ternary gas system with the same proportion of N_2_.

When we combine the simulation data and substitute Eq. (2) into Eq. (1), the relationship between the adsorption amount and adsorption potential energy of multi-component gases at different temperatures can be calculated, which is illustrated in Fig. 7.

Figure 7a and b demonstrate that by injecting the same proportion of CH_4_ and N_2_ into the system, the CO_2_ adsorption increases rapidly at the temperature of 263.15 K. This phenomenon occurred because of the increased CO_2_ concentration in the system resulting from the increase in CO_2_ injection in the system from 1 to 3%. Because of the adsorption advantage of CO_2_ itself over CH_4_ and N_2_, the relative adsorption of CO_2_ rises rapidly. When the system temperature increases from 263.15 to 283.15 K, the CO_2_ adsorption rapidly decreases because CO_2_ is more temperature-sensitive than CH_4_ and N_2_. The adsorption levels off when the temperature increases from 283.15 to 313.15 K. This is because the adsorption decreases with increasing temperature, which is consistent with the conclusion of the single-component gas adsorption. The adsorption potential energy of CO_2_ increases from 8.78 to 11.35 kJ/mol, that of CH_4_ from 9.79 to 12.56 kJ/mol, and that of N_2_ from 10.93 to 13.91 kJ/mol, indicating that the adsorption potential energy of each gas component increases with increasing temperature, and the adsorption potential energy of the gas components follows the order: CO_2_ < CH_4_ < N_2_. As shown in Fig. 7a, CO_2_ adsorption decreases from 1.01 to 0.24 mmol/g, CH_4_ adsorption decreases from 0.48 to 0.16 mmol/g, and N_2_ adsorption drops from 0.31 to 0.13 mmol/g. As shown in Fig. 7b, CO_2_ adsorption decreases from 0.85 to 0.22 mmol/g, CH_4_ adsorption decreases from 0.47 to 0.13 mmol/g, N_2_ adsorption decreases from 0.31 to 0.13 mmol/g, and the adsorption amounts of the gas components are in the order of CO_2_ > CH_4_ > N_2_. Therefore, the adsorption amount has a negative correlation with the adsorption potential energy, i.e. the quantity of gas adsorbed reduces as the adsorption potential energy increases. From a thermodynamic perspective, anthracite adsorbs the most CO_2_, followed by CH_4_ and N_2_. This phenomenon occurs because a rise in temperature increases the thermal movement of the solid surface molecules, weakening the intermolecular interaction forces and, consequently, reducing the surface energy. Thus, the CO_2_, CH_4_, and N_2_ molecules are not easily adsorbed on the surface of the coal molecules, which reduces the adsorption amount.

#### Potential energy distribution

**Figure 8:**
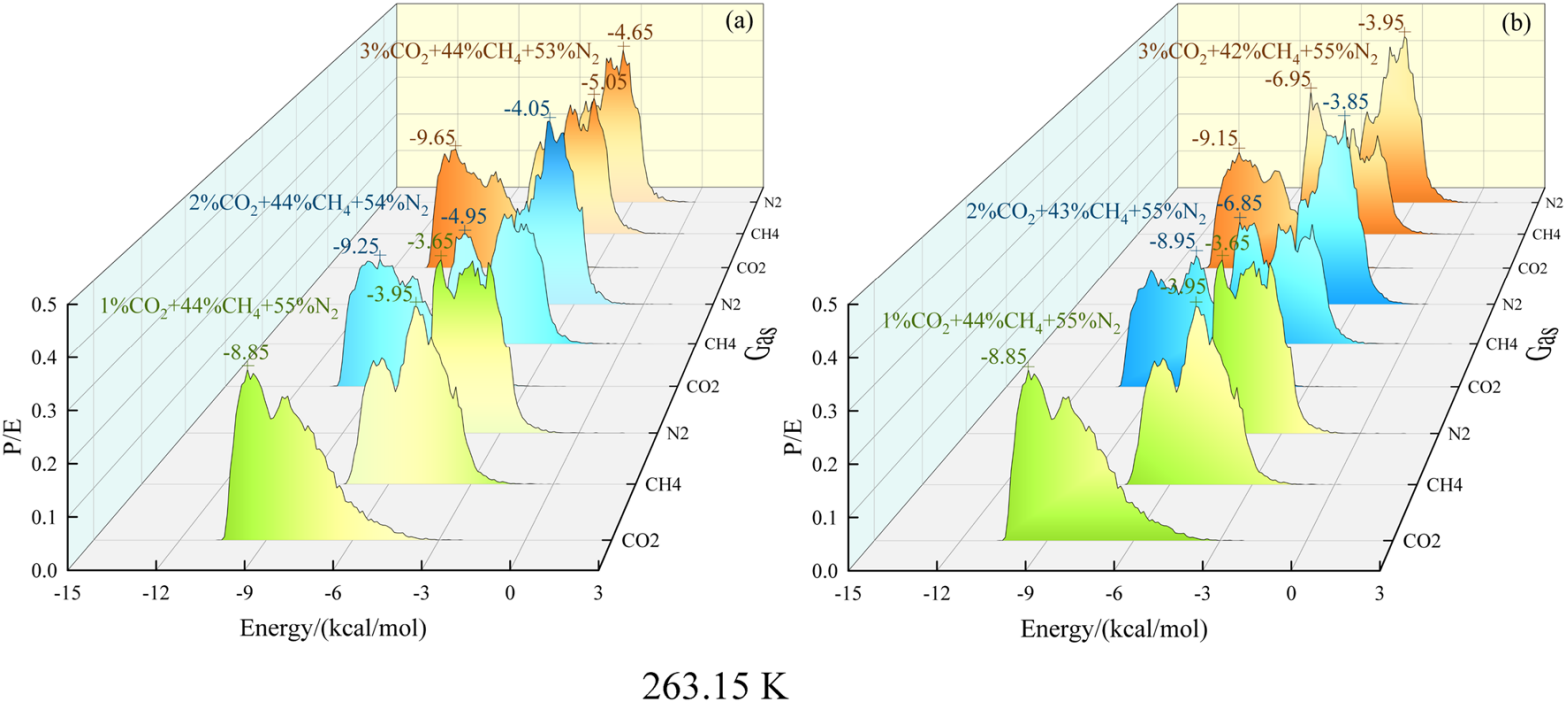
Potential energy distribution of multi-component gas. (**a**) and (**b**) are the potential energy distributions analyzed in the system of CH_4_ and N_2_ in the same proportions, with the initial ratio of CO_2_, CH_4_, and N_2_ all being 1:44:55.

The potential energy distribution of the anthracite adsorption of CO_2_, CH_4_, and N_2_ was obtained through a simulation, and the relationship between the preferential adsorption potential and the amount of gas injected was analyzed. The results are illustrated in Fig. 8 for the pressure of 0.1 MPa and temperature of 263.15 K. The data on the initial potential energy distribution of the gases are shown in Table 5.

**Table 5:** Potential energy distribution of initial adsorbates injected with equal proportions of CH_4_ (a) and equal proportions of N_2_ (b) in a ternary gas system.

No	Initial adsorbate potential for injection of equal proportions of CH_4_/(kcal/mol)			No	Initial adsorbate potential for injection of equal proportions of N_2_/(kcal/mol)		
	CO_2_	CH_4_	N_2_		CO_2_	CH_4_	N_2_
1	− 10.65	− 7.45	− 6.05	1	− 10.75	− 7.65	− 6.05
2	− 10.85	− 7.75	− 6.15	2	− 10.95	− 7.75	− 6.15
3	− 10.95	− 7.85	− 6.35	3	− 11.05	− 7.95	− 6.25

Adsorption energy is the energy produced during adsorption. Molecules decelerate and eventually stop at the surface of the adsorption medium during adsorption, which releases some energy. Thus, the greater the absolute value of the adsorption energy, the greater the intermolecular interactions and preferential adsorption.

A comparison of (a) and (b) in Fig. 8 shows that for different injection ratios of CO_2_, CH_4_, and N_2_ into the system, the absolute magnitude of the potential energy peak of CO_2_ increases with the increasing quantity of gas injected the system. In the two gas systems with certain CH_4_ and N_2_ ratios, the potential energy peak of the optimal adsorption site of CO_2_ decreases from − 8.85 kcal/mol to − 9.65 and − 9.15 kcal/mol, respectively. The potential energy peak of the optimal adsorption site of CH_4_ decreased from − 3.95 kcal/mol to − 5.05 and − 6.95 kcal/mol, respectively. The potential energy peak of the optimal adsorption site of N_2_ decreased from − 3.65 kcal/mol to − 4.65 and − 3.95 kcal/mol, respectively. A comparison of (a) and (b) shows that the optimal adsorption site for injecting the same proportion of CH_4_ into the system is higher than that of N_2_; thus, the preferential adsorption potential of CO_2_ is greater than that of CH_4_, and that of CH_4_ is greater than that of N_2_. This result is consistent with the order of magnitude of the amount of the three gases adsorbed. The absolute values of the potential energy peaks for both CH_4_ and N_2_ showed a rising pattern with the increase in CO_2_ injection; hence, CO_2_ can promote the adsorption behavior of CH_4_ and N_2_ on anthracite.

## Conclusions

1. The adsorption behavior of the single-component gas system is in accordance with Langmuir’s law of adsorption, and the values of adsorption constants k_1_, k_2_ are generally negatively correlated with temperature. From the perspective of the adsorption rate, CO_2_, CH_4_, and N_2_ attained the fastest adsorption rate in the single-component gas adsorption system at temperatures of 263.15 K, 273.15 K, 283.15 K, 293.15 K, 303.15 K, and 313.15 K, as well as pressures in the range of 0.01 to 2 MPa. In different pressure ranges, the adsorption rate of CO_2_ increased with increasing temperature because adsorption is typically exothermic, and when the adsorption process has not reached equilibrium, elevated temperatures accelerate the rate of adsorption. The adsorption amount was positively correlated with pressure and negatively correlated with temperature, and the adsorption amounts were in this order of magnitude: CO_2_ > CH_4_ > N_2_.
2. In the multi-component gas system, at the same pressure, the proportion of the relative adsorption amount of CO_2_ in the system increased from 1/3 to 2/3 with the increase in CO_2_ injection from 1 to 3%, whereas the relative adsorption of CH_4_ and N_2_ decreased. The relative CO_2_ adsorption was positively correlated with the amount of gas injected but negatively correlated with temperature. The relative adsorption amounts were in this order of magnitude: CO_2_ > CH_4_ > N_2_.
3. In the multi-component gas system, under identical pressures, the adsorption potential energy of CO_2_, CH_4_, and N_2_ at 263.15 K was 8.78 kJ/mol, 9.79 kJ/mol, and 10.93 kJ/mol, respectively, with the values increasing with increasing temperature. The adsorption potential energy of CO_2_, CH_4_, and N_2_ was positively correlated with temperature and negatively correlated with the adsorption amount. The adsorption potential energy of the three gases was in this order of magnitude: CO_2_ < CH_4_ < N_2_.
4. In the multi-component gas system, at the same temperature and pressure, the potential energy peaks of CO_2_, CH_4_, and N_2_ were − 8.85 kcal/mol, − 3.95 kcal/mol, and − 3.65 kcal/mol, respectively, and the absolute values at the optimal adsorption sites were in this order of magnitude: CO_2_ > CH_4_ > N_2_. The absolute values of the potential energy peaks of the optimal adsorption sites for CH_4_ and N_2_ increased as the injection of CO_2_ into the system increased. Furthermore, the preferential adsorption potentials followed the order of CO_2_ > CH_4_ > N_2_.

## Data Availability

All data supporting the findings of this study are available from the corresponding author Jiaxing Lin upon request.
